# The Contribution of Chemokines and Migration to the Induction of Central Tolerance in the Thymus

**DOI:** 10.3389/fimmu.2015.00398

**Published:** 2015-08-07

**Authors:** Zicheng Hu, Jessica Naomi Lancaster, Lauren I. R. Ehrlich

**Affiliations:** ^1^Ehrlich Laboratory, Department of Molecular Biosciences, Institute for Cellular and Molecular Biology, The University of Texas at Austin, Austin, TX, USA

**Keywords:** thymus, negative selection, central tolerance, chemokine receptors, thymocyte migration

## Abstract

As T cells develop, they migrate throughout the thymus where they undergo essential bi-directional signaling with stromal cells in distinct thymic microenvironments. Immature thymocyte progenitors are located in the thymic cortex. Following T cell receptor expression and positive selection, thymocytes undergo a dramatic transition: they become rapidly motile and relocate to the thymic medulla. Antigen-presenting cells (APCs) within the cortex and medulla display peptides derived from a wide array of self-proteins, which promote thymocyte self-tolerance. If a thymocyte is auto-reactive against such antigens, it undergoes either negative selection, via apoptosis, or differentiation into the regulatory T cell lineage. This induction of central tolerance is critical for prevention of autoimmunity. Chemokines and adhesion molecules play an essential role in tolerance induction, as they promote migration of developing thymocytes through the different thymic microenvironments and enhance interactions with APCs displaying self-antigens. Herein, we review the contribution of chemokines and other regulators of thymocyte localization and motility to T cell development, with a focus on their contribution to the induction of central tolerance.

## Introduction: Coordination of T Cell Development with Intrathymic Localization

**Figure 1:**
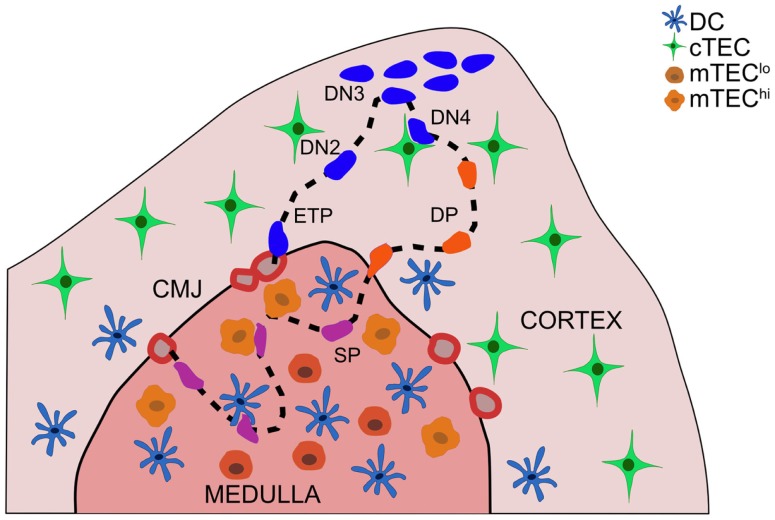
**Thymocyte migration through distinct thymic microenvironments occurs in an ordered fashion, enabling appropriate interactions with stromal cells**. Thymocyte progenitors enter the thymus through vessels at the cortico-medullary junction (CMJ). ETPs (CD3^−^CD4^−^CD8^−^c-Kit^+^CD44^+^CD25^−^) integrate cTEC-derived signals in the cortex near the CMJ, which promote survival and T-lineage-commitment. DN2 (CD3^−^CD4^−^CD8^−^c-Kit^+^CD44^+^CD25^+^) thymocytes migrate into the mid-cortex, as they rearrange TCRβ chain genes. Subsequent DN3 (CD3^−^CD4^−^CD8^−^c-Kit^−^CD44^−^CD25^+^) thymocytes that pass the β-selection checkpoint proliferate at the sub-capsule, and differentiate through a DN4 (CD3^−^CD4^−^CD8^−^c-Kit^−^CD44^−^CD25^−^) stage to become DP (CD4^+^CD8^+^) thymocytes. DP cells, which rearrange TCRα chain genes, are localized throughout the cortex, with a bias toward the medulla. Interactions with cTECs induce positive selection of DP cells expressing TCRs with low avidity for self-peptide:MHCs. Auto-reactive DP thymocytes can be negatively selected in the cortex. Positively selected DP cells begin to migrate rapidly and enter the thymic medulla, guided by chemokine gradients, as they differentiate into SP thymocytes. SP cells rapidly scan mTECs and DCs during their 4–5-day residence time in the medulla to encounter a wide array of self-peptides, which induce auto-reactive cells to undergo apoptosis or diversion into the Treg lineage. Mature SP thymocytes egress from the thymus through blood vessels in the CMJ.

Thymocytes migrate through distinct thymic microenvironments at discrete stages of differentiation in order to receive essential signals from surrounding stromal cells that govern further differentiation and selection (1, 2) (Figure 1). Early thymocyte progenitors (ETP) localize to the cortical side of the cortico-medullary junction (CMJ). As they commit to the T-lineage, thymocytes migrate into the mid-cortex, where they rearrange T cell receptor (TCR) β chain genes (3). Cells that successfully express TCRβ pass the β-selection checkpoint, and undergo proliferation and differentiation near the sub-capsule. Subsequent double positive (DP) thymocytes are localized throughout the cortex, where they rearrange TCRα chain genes. DP cells that receive weak TCR signals in the cortex undergo positive selection, promoting survival and differentiation of self-MHC-restricted single positive (SP) cells. SP thymocytes migrate into the medulla, where auto-reactive cells receiving strong TCR signals are culled from the repertoire or diverted into the regulatory T cell (Treg) lineage. We will review migratory and adhesion cues governing localization and cellular interactions of differentiating thymocytes and stromal cell subsets, with an emphasis on signals that promote central tolerance. Recent advances and open questions will be highlighted.

## Migration and Stromal Interactions during Early Stages of Thymocyte Differentiation

Common lymphoid progenitors or their immediate progeny enter the thymus through vasculature at the CMJ (4), and subsequently give rise to developing T cells (5–7). Transmigration through the endothelium is initiated by selectin-mediated rolling (P-selectin), followed by firm adhesion via integrins (α4β1 and αLβ2) in concert with chemokine receptor signaling (CCR9, CCR7) (8–12). Within the thymus, cortical thymic epithelial cells (cTECs) provide IL7, SCF, and DLL4, which are indispensable for survival, differentiation, and T-lineage-commitment of thymocyte progenitors (13–15). ETP and double negative 2 (DN2) cells express CXCR4, which promotes chemotaxis toward cTEC-derived CXCL12 (16–20). Cortical thymocytes also express integrin α4β1, which binds VCAM-1 on cTECs. CXCR4 deficiency or impaired VCAM-1 adhesion inhibits thymocyte differentiation and migration from the CMJ to the mid-cortex (20–23). It remains to be determined how CCR7 promotes both thymic entry of progenitors into the cortex, and medullary accumulation of SP thymocytes (see below). As ETP do not express CCR7, rapid downregulation of CCR7 following thymic entry may enable cortical progenitor localization.

## Migration and Stromal Interactions of Thymocytes Undergoing β-Selection

DN3 cells completing TCRβ rearrangements localize to the outer capsule (4). In addition to pre-TCR signals, activation of CXCR4 (24), NOTCH-1 (13, 25), and IL7R via cTEC ligands (1, 26) are required for differentiation and expansion at the β-selection checkpoint. The consequences of or signals governing sub-capsular localization of proliferating post-β selection cells remain to be elucidated (3). CCR9 is first expressed at the DN3 stage, and DN3 through DP thymocytes migrate toward CCL25, expressed by cTECs (17, 18, 27). Deficiency or overexpression of CCR9 prevents DN3 accumulation at the sub-capsule (12, 28, 29). However, a role for CCR9 in sub-capsular localization is hard to reconcile with the distribution of CCL25 throughout the cortex (30) or the CCR9-responsiveness of DP cells, which are also present throughout the cortex (17, 18). Moreover, we have shown that pre-positive selection DP thymocytes, which are CCR9 responsive, accumulate near the medulla, not the sub-capsule (31). Thus, signals governing DN3 accumulation at the sub-capsule remain to be identified.

## Migratory Cues Governing Localization and Stromal Interactions of DP Thymocytes

We speculate that plexinD1 may promote rapid motility and peri-medullary accumulation of pre-positive selection DP cells (31). Sema3e, a soluble plexinD1 ligand produced in the medulla, inhibits CCR9-mediated chemotaxis, releases integrin α4β1 catch bonds, and is required for medullary localization of post-positive selection thymocytes (32–34). However, pre-positive selection DP cells also express plexinD1; thus, DP cells that reach the peri-medullary cortex, perhaps through random migration (35), would encounter Sema3e, potentially diminishing CCR9-mediated migration back into the cortex, and relaxing adhesion to VCAM-1 on cTECs, thus increasing motility. Recent studies demonstrate that GIT2, which modulates actin reorganization during cellular migration, also promotes rapid migration of cortical thymocytes (36). GIT2 and plexinD1 may coordinately enhance the ability of DP cells to efficiently scan cTECs for positively selecting ligands, which is consistent with the impaired positive selection in *Git2^−/−^* mice (36). Future studies may resolve the roles of plexinD1 and GIT2 in localization, migration, and positive selection of pre-positive selection DP thymocytes.

## Migratory Cues and APCs Governing Cortical Negative Selection

Although the medulla is a critical environment for negative selection, there is mounting evidence that the cortex promotes deletion of a significant number of auto-reactive thymocytes. Thymocytes undergoing negative selection were recently quantified using *Bim^−/−^*; Nur77^GFP^ mice (37), in which apoptotic cells survive due to deficiency in the Bcl2 family member Bim, and GFP levels reflect TCR signal strength, enabling quantification of cells that should have been deleted due to strong TCR signaling. In the absence of Bim, GFP^+^ DP and GFP^+^ SP cellularity was increased, demonstrating that negative selection occurs in both compartments. Interestingly, the increase in GFP^+^ DP cells was up to threefold higher than GFP^+^ SPs (37), suggesting that over 90% of positively selected DP thymocytes are fated for cortical deletion (38). Another study analyzed Helios levels in *Bim^−/−^* mice to estimate that 55% of TCR-signaled thymocytes are deleted at the DP stage (39). Together, these studies indicate that the majority of negative selection occurs in DP cells, raising the question of which cortical antigen-presenting cells (APCs) promote central tolerance.

Cortical thymic epithelial cells are uniquely responsible for inducing positive selection (40); however, their role in negative selection remains ambiguous. Early studies established that thymic grafts transplanted into allogeneic athymic hosts were tolerated by host-derived T cells (41–45). Developing T cells are likely tolerized to graft-derived peptide:MHCs expressed by medullary TECs (mTECs) or DCs, which does not clarify whether cTECs induce negative selection. To address this, transgenic mice were developed in which MHC-I (46) or MHC-II (47) was expressed exclusively on cTECs. cTECs in these mice induced positive selection of CD8SP or CD4SP thymocytes, respectively, but could not tolerize polyclonal thymocytes. In light of the essential contribution of mTECs to negative selection against diverse self-antigens (see below), these findings do not resolve whether cTECs induce deletion of some auto-reactive clones. Expression of model antigens uniquely in cTECs resulted in deletion of TCR transgenic thymocytes, indicating that cTECs can mediate negative selection (48). However, when a TCR transgene was more faithfully expressed at the later DP stage in the HY^CD4^ model, cTECs expressing the cognate antigen induced TCR activation, but not apoptosis of auto-reactive DP cells (49). Thus, cTECs can clearly activate auto-reactive TCRs, but their ability to mediate deletion remains uncertain.

DCs have emerged as likely mediators of cortical negative selection. DCs express high levels of costimulatory and MHC molecules, enabling strong TCR activation (50). Strikingly, in a model of cortical negative selection, thymocytes undergoing apoptosis were localized adjacent to cortical DCs, and negative selection was impaired when DCs were conditionally ablated (49). The migratory cues that promote thymocyte:DC interactions during cortical negative selection have yet to be elucidated. Cortical DCs accumulate near vasculature, where CCR7 ligands are presented (30, 51, 52). Thymic DCs undergo CCR7-mediated chemotaxis (53), suggesting CCR7 may position DCs near cortical blood vessels. CCR7 was also postulated to induce cortical thymocytes to associate with DCs under positively selecting conditions (52). However, CCR7 is not up-regulated until the SP stage (54), when thymocytes home to the medulla, and CCR7 was dispensable for cortical deletion in the HY^cd4^ model (49). Thus, CCR7 signaling may position cortical DCs near vasculature, but is unlikely to promote thymocyte:DC interactions during cortical negative selection. CCR2 also contributes to cortical DC positioning, as it recruits migratory DCs to perivascular spaces in the cortex to induce deletion against blood-borne antigens (55, 56). CCX-CKR1 (CCRL1) regulates bioavailability of CCL19, CCL21, and CCL25, but its expression by cTECs and impact on tolerance are currently controversial (57, 58). Further investigation is needed to elucidate the contributions of APCs and migratory cues governing cortical negative selection.

## Migration of Post-Positive Selection Thymocytes into the Medulla

The migration of post-positive selection thymocytes into the medulla is critical for the induction of central tolerance. If the medulla does not develop, or thymocytes cannot accumulate therein, negative selection is impaired, and autoimmunity arises (59–64). Only positively selected thymocytes gain access to the medulla (31); recent evidence suggests CXCR4 is responsible for cortical retention of DP cells (65). Following positive selection, thymocytes migrate much more rapidly (12–16 μm/min post-selection versus 6–8 μm/min pre-selection) and undergo chemotaxis toward the medulla (31, 66, 67). It is commonly assumed that thymocytes enter the medulla at the SP stage. However, plexinD1 deficiency results in relocalization of CD69^+^ cells from the medulla into the cortex, suggesting post-positive selection CD69^+^ DP cells may enter the medulla (32, 33). Furthermore, the kinetics of medullary entry after positive selection, compared to the timing of differentiation from the DP to SP stage indicates that CD69^+^ DP cells enter the medulla (68). Thus, positive selection likely induces rapid thymocyte medullary entry; further studies are required to determine if and how CD69^+^ DP cells overcome cortical retention to enter the medulla.

**Figure 2:**
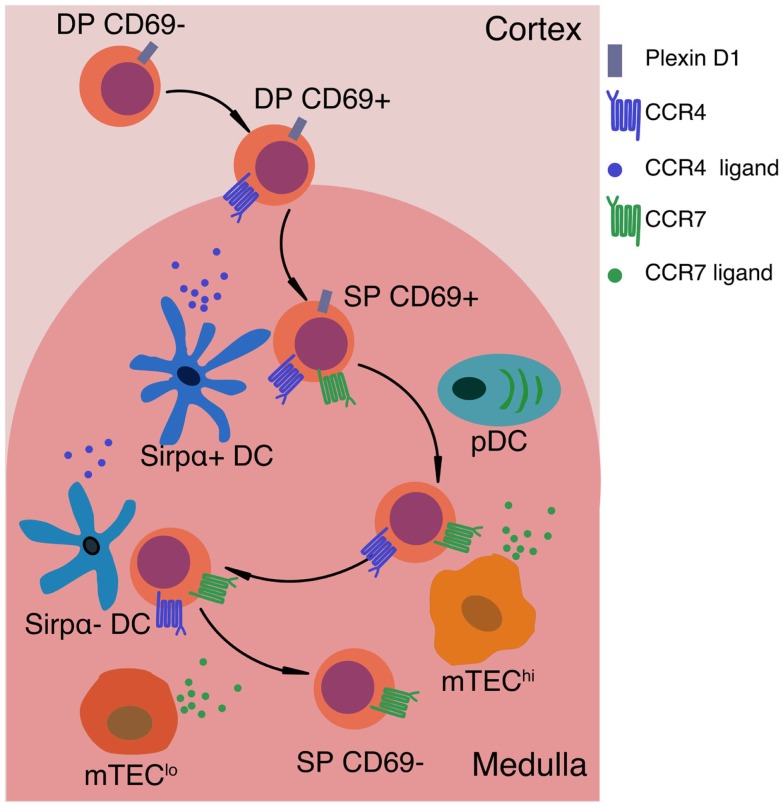
**Signals that impact motility and localization of positively selected thymocytes**. After positive selection, thymocytes up-regulate CD69 and the chemokine receptor CCR4. CCR4 ligands are expressed by medullary DCs, thus creating a chemotactic gradient that may promote medullary entry of post-positive selection thymocytes. PlexinD1 is expressed on DP and immature SP thymocytes, and may also promote medullary entry by inhibiting responses to cortical chemokines and releasing thymocytes from tight interactions with cTECs. As SP cells mature, they up-regulate CCR7, promoting chemotaxis toward the gradient of CCR7 ligands produced by mTEC^lo^ (CD80^lo^MHC-II^lo^) and mTEC^hi^ (CD80^+^MHC-II^hi^) cells. CCR7 signaling is critical for maintaining SP thymocytes within the medulla. In the absence of CCR7, SP cells do not undergo efficient negative selection against TRAs. Expression of CCR7 and CCR4 on SP thymocytes may also promote chemokinesis, or rapid motility of SP thymocytes, as well as efficient interactions with the two main subsets of medullary APCs, mTEC^hi^ cells and DCs, respectively. Thus, chemokine-guided migration likely impacts multiple aspects of SP motility and cellular interactions that are required to ensure SP thymocytes efficiently scan numerous medullary APCs to encounter the full array of self-antigens that induce central tolerance.

The chemokine receptor CCR7 is critical for thymocyte localization in the medulla (31, 51). CCR7 is expressed by SP thymocytes (51, 54, 69, 70), while the ligands CCL19 and CCL21 are expressed by mTECs (71). In mice deficient for CCR7 or its ligands, medullary accumulation of SP cells is diminished, negative selection is impaired, and autoimmunity ensues (59, 60). Although CCR7 is required for SP chemotaxis toward the medulla and accumulation therein, *Ccr7^−/−^* SP cells enter and migrate within the medulla (31). In contrast, SP medullary entry is abrogated by pertussis toxin (31, 69), which blocks signaling through Gα_i_-associated G protein coupled receptors (GPCRs), including chemokine receptors. Thus, other GPCRs must contribute to thymocyte medullary localization. We speculate that CCR4 may contribute to medullary entry. CD69 + DP and CD69 + CD4SP thymocytes express CCR4 (54, 69) and undergo chemotaxis toward the ligands CCL17 and CCL22 (17), which are expressed in the medulla (18, 72). CCR4 is up-regulated early after positive selection, while CCR7 is expressed on more mature SP cells (54), suggesting differential roles in guiding thymocytes into the medulla. CCR4 may be responsible for initial medullary entry of post-positive selection cells, while CCR7 may promote retention of maturing SPs (Figure 2). Future studies are required to address the relative contributions of CCR4 and other GPCRs to medullary entry and central tolerance, though a recent study did not identify a role for CCR4 in these processes (54).

## APCs Governing Medullary Negative Selection and Treg Generation

Once SP thymocytes migrate into the medulla, they encounter heterogeneous APCs that enforce self-tolerance (Figure 2). Medullary APCs display peptides derived from a wide array of tissue-restricted antigens (TRAs), proteins otherwise expressed by peripheral tissues. mTEC^hi^ cells express high levels of CD80 and MHC-II, as well as the transcriptional regulator AIRE, which induces mTEC differentiation and expression of diverse TRAs that were previously epigenetically silenced (73–81). AIRE-dependent expression of such TRAs is essential for the induction of central tolerance in mice and humans (82–87). Medullary DCs also contribute to negative selection; they can be divided into intrathymically derived Sirpα^−^ conventional DC (cDC), migratory Sirpα^+^ cDC, and plasmacytoid DCs (pDC) (88, 89). Other APCs, such as B cells, may also contribute to negative selection (90–92), but are not discussed here.

Several experimental models indicate that mTECs can directly present peptide:MHCs to mediate negative selection and Treg induction. Negative selection against model self-antigens was intact following ablation of DCs or MHC-II expression on hematopoietic cells, demonstrating that mTECs can be sufficient to mediate negative selection (93, 94). Furthermore, miRNA-mediated reduction of MHC-II expression in mTECs resulted in diminished negative selection of TCR transgenic thymocytes to a model TRA, demonstrating that direct antigen presentation by mTECs is required for deletion in some cases (95). Direct presentation of TRAs by mTECs can also induce Treg differentiation (96). While endogenous proteins in mTECs will naturally access the MHC-I processing and presentation pathway, presentation on MHC-II is facilitated by macroautophagy, which is required for central tolerance (97). Thus, mTECs have an intrinsic capacity to present diverse self-antigens to mediate central tolerance of CD4SP and CD8SP cells.

DCs are also critical for thymic central tolerance. DC ablation in a CD11c-DTA model resulted in impaired negative selection and fatal autoimmunity (98). MHC-II ablation on hematopoietic cells impaired both Treg induction and negative selection against serum-borne and soluble TRAs (99–101). Sirpα^+^ cDC and pDC can acquire peripheral antigens and traffic them to the thymus to induce negative selection (102, 103). Also, in some models of mTEC-expressed TRAs, DCs isolated from the thymus stimulate TRA-specific T cells specific more efficiently than mTECs themselves, indicating that antigens are transferred efficiently from mTECs to DCs to mediate deletion (104). Transfer of model TRAs from mTECs to DCs can be AIRE-dependent and required for negative selection (99, 104). The mechanisms of antigen transfer between mTECs and DCs remain to be elucidated. mTECs may secrete or release antigen in vesicles; DCs may acquire antigen by endocytosis of apoptotic mTECs (105); or peptide:MHC complexes may be acquired by DCs from mTEC cell membranes (104, 106). Thus, the heterogeneous thymic DC compartment promotes central tolerance against peripheral, blood-borne, and mTEC-derived self-antigens.

While both mTECs and DCs induce tolerance to some antigens, their relative contributions to central tolerance of polyclonal thymocytes have been difficult to ascertain. Using TCR repertoire analysis of Treg and naïve T cells, Perry et al. recently compared the impact of restricting antigen presentation to DCs versus mTECs (107). mTECs and DCs mediated negative selection of non-overlapping TCRs, and DCs deleted about threefold more TCRs. These findings are in keeping with studies showing that both subsets are important for negative selection. Furthermore, both mTECs and DCs induced Treg differentiation. AIRE was critical for negative selection and Treg induction of lower frequency TCRs, and the Sirpα^−^ subset of cDC was required for AIRE-dependent Treg generation (107). Importantly, this study compared the effects of diminished MHC-II expression on mTECs with ablated MHC-II expression on DCs, and may thus underestimate the relative contribution of mTECs to central tolerance. Nonetheless, it is clear that complete central tolerance will require efficient thymocyte interactions with both mTECs and DCs.

## Migratory Cues Promoting Medullary Central Tolerance

Given that DCs acquire TRAs from mTECs, it is likely DCs must localize near mTECs to mediate efficient central tolerance. Consistent with this, XCR1, which is expressed on Sirpα^−^cDC, was required for localization of cDC to the center of the medulla (53). In *Xcl1^−/−^* mice, Treg cellularity was diminished, the TCR repertoire was altered, and autoimmune manifestations occurred, indicating that medullary localization of Sirpα^−^cDC is required for central tolerance (53). This suggests a model in which XCR1 promotes direct apposition of Sirpα^−^DCs with mTECs for TRA acquisition. Sirpα^+^ cDC and pDC, which carry peripheral antigens into the medulla, migrate into the thymus through vasculature in a P-selectin, VLA4, and GPCR-dependent manner (103). CCR9 is required for thymic entry of pDC, but the corresponding GPCR for Sirpα^+^ cDC has not been identified (102). Although thymic DCs express CCR7 and migrate toward CCR7 ligands, *Ccr7^−/−^* DCs localize properly within the medulla (53). Thus, signals required for medullary localization of Sirpα^+^ DCs and pDCs remain to be identified.

SP thymocytes were recently estimated to have a medullary residence time of ~4–5 days (108), shorter than the previous estimate of ~12 days (109), and each AIRE-dependent TRA is expressed on only 1–3% of AIRE^+^mTEC^hi^ cells (74, 110). Thus, thymocytes must rapidly scan multiple mTECs and DCs to encounter the full spectrum of medullary self-antigens that promote central tolerance. Chemokines can promote lymphocyte chemokinesis (111), and CCR7 has been shown to promote rapid motility of SP thymocytes (31). Fast SP migration is also dependent on MST1, which promotes integrin-mediated binding of SP thymocytes to ICAM1 in the context of CCL21 (112). It remains to be determined whether other chemokine signals are required for rapid motility of SP thymocytes.

It remains to be established whether interactions between thymocytes and medullary APCs are driven by chemotaxis toward APCs or random encounters due to fast SP motility. Several studies suggest chemokines may facilitate T cell:APC interactions in secondary lymphoid organs. Using microspheres releasing CCL19 and CCL21, a recent study demonstrated that when sources of CCR7 ligands were interspersed, T cells hopped between microspheres, potentially facilitating antigen sampling (113). Both CCR4 and CCR7 have been implicated in promoting T cell:APC interactions that drive naïve T cell activation (114, 115). Thus, CCR4 and CCR7 may also promote cellular interactions between SP cells and DCs and mTECs, respectively. Indeed, Mst1 was required for efficient interactions between SP cells undergoing negative selection and Aire^+^mTECs expressing a model TRA (112), suggesting that CCR7 may enhance adhesion between SP cells and mTECs via intergrin:ICAM1 interactions. Furthermore, CCR7 deficiency was recently shown to result in increased Treg cellularity (54), which may also reflect the contribution of CCR7 to avid APC interactions. Although the basis for the decision to undergo apoptosis versus Treg specification is not resolved, current models favor an avidity model in which the highest avidity TCR signals promote negative selection, while a range of slightly lower avidity signals promote Treg induction as well as negative selection (116, 117). Thus, if CCR7 promotes T cell:APC interactions, CCR7 deficiency might result in lower avidity interactions that favor Treg induction. The fact that CCR7 ligands are expressed by mTECs, while CCR4 ligands are expressed by DCs also raises the possibility that CCR7 and CCR4 promote interactions with mTECs and DCs, respectively (Figure 2). Further investigation will be required to elucidate the contribution of chemokines or other adhesion molecules to interactions with medullary APCs driving central tolerance.

## Areas for Future Investigation

Chemokine receptors and integrins promote migration and adhesion required for thymocyte:stromal interactions that drive T cell differentiation and selection. However, multiple localization and migration cues remain to be elucidated. We have not identified signals driving localization of DN3 thymocytes to the sub-capsule, accumulation of pre-selection DP cells near the medulla, or thymocyte:APC interactions during cortical negative selection. The identities of GPCRs other than CCR7 that promote medullary entry and APC interactions remain to be determined. We are just beginning to appreciate that localization of stromal cells is critical for thymocyte differentiation, and future studies will likely identify factors driving proper stromal organization. Thus, many open questions remain regarding the localization and adhesion cues that promote differentiation of a fully functional and self-tolerant T cell compartment.

## Conflict of Interest Statement

The authors declare that the research was conducted in the absence of any commercial or financial relationships that could be construed as a potential conflict of interest.
